# Supplementary material for: Self-Assembled TiN-Metal Nanocomposites Integrated on Flexible Mica Substrates towards Flexible Devices
Source: Sensors (Basel). 2024 Jul 26;24(15):4863. doi: 10.3390/s24154863 (PMC11314696; doi:10.3390/s24154863)
Supplement: Supplementary file 1 [file sensors-24-04863-s001.zip › sensors-3002945-supplementary.pdf]

Supplementary Information

Self-assembled TiN-metal nanocomposites integrated on flexible mica substrates towards flexible devices

Juncheng Liu[1], Yizhi Zhang[1], Hongyi Dou[1], Benson Kunhung Tsai[1], Abhijeet Choudhury[1], and Haiyan Wang[1,2]*

[1] School of Materials Engineering, Purdue University, West Lafayette, Indiana 47907, United States

[2] School of Electrical and Computer Engineering, Purdue University, West Lafayette, Indiana 47907, United States

Matrix	Nanopillar	Substrate	Transmittance peaks (nm)	Coercivity [IP, OP] (Oe)	Ref
TiN	NiO	MgO	500, 620	-, 19.157	47
TiN	NiO(core)+Au(shell)	MgO		40.91, 13.81	53
TiN	Au	MgO	400	-	16
TiN & BTO	Au	MgO	490, 600	-	43
TiN	Ag	MgO	374, 350	-	50
TiN	Au-Ni	Mica	470, 515	102.5, 144.3	This work

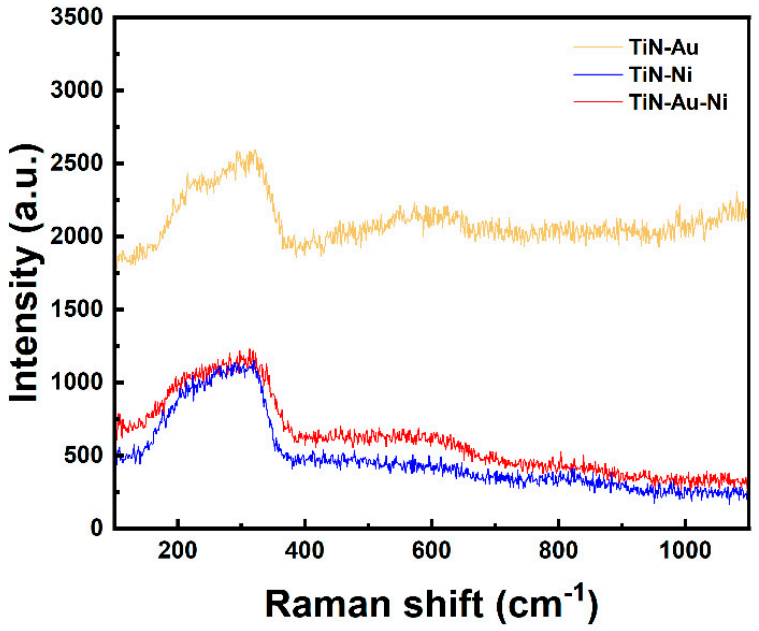

Figure S1. Raman measurement of the TiN-Au, TiN- Ni and TiN-Au-Ni film.
